# Low Dose of Penfluridol Inhibits VEGF-Induced Angiogenesis

**DOI:** 10.3390/ijms21030755

**Published:** 2020-01-23

**Authors:** Suyash Srivastava, Fatema Tuz Zahra, Nehal Gupta, Paul E. Tullar, Sanjay K. Srivastava, Constantinos M. Mikelis

**Affiliations:** 1 Department of Biomedical Sciences, School of Pharmacy, Texas Tech University Health Sciences Center, Amarillo, TX 79106, USAfatema.zahra@ttuhsc.edu (F.T.Z.); nengupta2890@gmail.com (N.G.); 2 Department of Pharmaceutical Sciences, School of Pharmacy, Texas Tech University Health Sciences Center, Amarillo, TX 79106, USA; 3 Department of Immunotherapeutics and Biotechnology, and Center for Tumor Immunology and Targeted Cancer Therapy, Texas Tech University Health Sciences Center, Abilene, TX 79601, USA; 4 Department of Obstetrics and Gynecology, School of Medicine, Texas Tech University Health Sciences Center, Amarillo, TX 79106, USA; paul.tullar@ttuhsc.edu

**Keywords:** penfluridol, angiogenesis, breast cancer

## Abstract

Metastasis is considered a major burden in cancer, being responsible for more than 90% of cancer-related deaths. Tumor angiogenesis is one of the main processes that lead to tumor metastasis. Penfluridol is a classic and commonly used antipsychotic drug, which has a great ability to cross the blood–brain barrier. Recent studies have revealed that penfluridol has significant anti-cancer activity in diverse tumors, such as metastatic breast cancer and glioblastoma. Here, we aim to identify the effect of low doses of penfluridol on tumor microenvironment and compare it with its effect on tumor cells. Although low concentration of penfluridol was not toxic for endothelial cells, it blocked angiogenesis in vitro and in vivo. In vitro, penfluridol inhibited VEGF-induced primary endothelial cell migration and tube formation, and in vivo, it blocked VEGF- and FGF-induced angiogenesis in the matrigel plug assay. VEGF-induced VEGFR2 phosphorylation and the downstream p38 and ERK signaling pathways were not affected in endothelial cells, although VEGF-induced Src and Akt activation were abrogated by penfluridol treatment. When cancer cells were treated with the same low concentration of penfluridol, basal Src activation levels were mildly impaired, thus impacting their cell migration and wound healing efficiency. The potential of cancer-induced paracrine effect on endothelial cells was explored, although that did not seem to be a player for angiogenesis. Overall, our data demonstrates that low penfluridol levels, similar to the ones clinically used for anti-psychotic conditions, suppress angiogenic efficiency in the tumor microenvironment.

## 1. Introduction

Breast cancer is the most common type of cancer among women worldwide, with an estimated number of about 43,000 deaths only in the United States in 2019 [1,2]. According to American Cancer Society, one out of eight women is expected to be affected by breast cancer in their lifetime in the United States, and the Centers for Disease Control and Prevention (CDC) have reported that breast cancer is the second leading cause of cancer death globally and the leading cause of cancer death among Hispanic women in 2019 [3]. Despite several treatment options and the improvement in the survival rate of breast cancer patients due to advancements in diagnostic procedures [4], breast cancer treatment still remains the major hurdle for breast cancer patients.

Angiogenesis is the formation of new blood vessels from pre-existing ones. It is essential during embryonic development and in certain conditions during adult life, such as wound healing, while imbalanced angiogenesis characterizes or deteriorates several pathological conditions, including cancer. Angiogenesis is regulated by the coordinated balance of growth factors, such as vascular endothelial growth factor (VEGF) and basic fibroblast growth factor (bFGF), and angiogenesis inhibitors [5,6]. A characteristic example of disruption of this coordinated balance is tumor-induced angiogenesis, which is responsible for primary tumor growth, formation of metastases, has been a significant research focus for several decades and has rendered anti-angiogenic therapy an important part of clinical oncology [7,8]. The newly formed tumor vessels are vital for tumor oxygenation and nutrient supply; however, they present significant morphological abnormalities, such as the chaotic networks that lack hierarchical arrangement, the absence of pericytes, the modified extracellular matrix and the large intercellular openings, enabling trans-endothelial migration and metastasis [9,10]. The inhibition of these characteristics and the normalization of the tumor vasculature is the latest focus on tumor vascular research. Tumor vessel normalization aims to increase tumor perfusion, increasing the efficacy of anti-cancer therapies and blocking the metastatic potential [11,12]. Cancer-induced angiogenesis blockade and tumor vessel normalization are major goals for cancer treatment and both are approached through targeting VEGF or the VEGFR signaling pathway [13,14].

VEGF is the main target of anti-angiogenic therapy. It is secreted at high levels from tumor cells and induces endothelial cell proliferation, migration, and tube formation [11,15,16]. VEGF binds to three receptor tyrosine kinases, VEGFR1, VEGFR2 and VEGFR3, which have overlapping but distinct expression patterns: VEGFR1 is highly expressed in monocytes and macrophages, VEGFR2 in vascular endothelial cells, and VEGFR3 in lymphatic endothelial cells. Although VEGFR1 has higher binding affinity with VEGF, its kinase activity is poor; it is not required for endothelial cell function and mostly acts as a negative regulator, blocking VEGF interaction with VEGFR2 [17,18]. VEGFR2 is the main VEGF receptor in the endothelial cells and most anti-angiogenic inhibitors either block VEGF-VEGFR2 interaction or inhibit VEGFR2 kinase activity. Main downstream targets activated by VEGFR2 are phospholipase Cγ (PLCγ), Src kinases, focal adhesion kinase (FAK), the PI3K-Akt pathway, the Rho family of monomeric G proteins and reactive oxygen species (ROS), which are responsible for VEGF-induced endothelial functions, such as angiogenesis (cell migration, proliferation, tube formation) and vascular permeability [18,19,20]. Tumor vasculature, as well as the vasculature of the surrounding tissue, is a part of the tumor microenvironment. Contrary to the genetic instability of the tumors, the tumor microenvironment, and in this case the endothelial cells, is a safe target for pharmacological intervention, independent of the driving mutations of each cancer cell type [21,22].

Penfluridol is a potent, long-acting oral antipsychotic drug long used for schizophrenia, acute psychosis, and Tourette syndrome [23,24,25,26]. Our previous studies have shown the anti-cancer efficacy of penfluridol against breast, brain, and pancreatic carcinoma [27,28,29,30]. However, there is no study on the effect of penfluridol on the tumor vascular microenvironment, especially in clinically relevant doses prescribed for schizophrenia and other psychotic disorders. This study aims to identify the effect of low doses of penfluridol on endothelial cell functions, highlighting an important parameter for anti-cancer research, and providing novel clinical implications of this historically safe and widely used drug.

## 2. Materials and Methods

### 2.1. Antibodies and Reagents

Endothelial cell growth supplement (ECGS) (Cat# 356006) and 5000 U/mL heparin solution (Cat# NDC#63739-920-11) were purchased from Corning (San Jose, CA, USA) and Hospira (Lake Forest, IL, USA) respectively. Chemicals, FBS (Cat# 10438026), DMEM (Cat# 11-995-073), 1X antibiotic-antimycotic solution (Cat# 15240-062) (all from GIBCO^TM^), 3-[4, 5-dimethylthiazol-2-yl]-2, 5-dimethyltetrazolium bromide (MTT) (Cat# AC15899) and hemoglobin assay kits (Cat# 50-489-219) were purchased from Fisher Scientific (Hampton, NH). The Halt Protease and Phosphatase Inhibitor Cocktail (Cat# PI78445) was purchased from Thermo Fisher Scientific (Waltham, MA, USA). Primary antibodies against phospho-VEGF receptor 2 (Tyr1175) (Cat# 2478), phospho-p38 (Thr180/Tyr182) (Cat# 4511), phospho-ERK (Cat# 9010), phospho-FAK (Tyr397) (Cat# 8556), phospho-Src (Tyr416) (Cat# 2101), phospho-AKT (Ser473) (Cat# 4060), β-actin (Cat# 3700), and β-tubulin (Cat# 2146) were purchased from Cell Signaling Technology (Beverly, MA, USA). Goat anti-rabbit secondary antibody (Cat# 4010-05, 1:50,000) was obtained from Southern Biotech (Birmingham, AL). Immobilon Western Chemiluminescent HRP substrate (Cat# WBKLS0500) and Immobilon P, a polyvinylidene difluoride membrane (Cat# IPVH304F0), were purchased from Millipore (Burlington, MA, USA). Human VEGF (Cat# SRP3182), murine VEGF (Cat# V4512), murine bFGF (Cat# SRP4038), penfluridol (Cat# P3371), SU1498 (Cat# SML1193), gelatin solution (Cat# S8636) and Medium 199 (Cat# M4530) were obtained from Sigma-Aldrich (St. Louis, MO, USA). Cultrex^®^ PathClear Reduced Growth Factor Basement Membrane Extract (Cat# 3433-010-01) was purchased from R&D Systems (Minneapolis, MN, USA). Sulforhodamine B (Cat# 80100) was purchased from Biotium (Fremont, CA, USA).

### 2.2. Cell Lines and Culture Procedures

Human umbilical vein endothelial cells (HUVECs) were isolated from human umbilical cords following Institutional Review Board (IRB)-approved protocol A15-3891 (Texas Tech University Health Sciences Center Institutional Review Board) and informed consent was obtained from all donors. HUVECs from at least three different donors were used for each experiment unless stated otherwise and we used between passages 1 and 6 for experiments. They were cultured in M199 medium, supplemented with 15% fetal bovine serum (FBS), 150 μg/mL Endothelial Cell Growth Supplement (ECGS), 5 U/mL heparin sodium and 1X antibiotic-antimycotic solution (EC complete medium). Human triple-negative breast carcinoma cell line MDA-MB-231 was purchased from ATCC. MDA-MB-231 cells were cultured in DMEM medium, supplemented with 10% FBS and 1X antibiotic-antimycotic solution (complete medium). All cells were maintained at 37 °C with 5% CO_2_ in a humidified environment, following standard protocols, and were regularly tested for mycoplasma contamination [31,32].

### 2.3. MTT Cytotoxicity Assay

Cytotoxicity of penfluridol on HUVECs was evaluated through the MTT (3-[4, 5-dimethylthiazol-2-yl]-2, 5-dimethyltetrazolium bromide) colorimetric assay, as described previously [33]. HUVECs were seeded on gelatin-coated 24-well plates at a density of 2 × 10^4^ cells/well and cultured in complete medium (500 μL/well). After 24 h of incubation at 37 °C with 5% CO_2_, they were treated with different concentrations of penfluridol (stock 5 mM in DMSO) for 48 h in complete medium. At the end of the incubation period, 50 μL of MTT stock (5 mg/mL in PBS) was added to each well and incubated for 2 h at 37 °C to allow the formation of dark blue formazan crystals in the metabolically active cells. The medium was removed, the cells were washed with PBS, and 100 μL of acidified isopropanol (0.33 mL HCl in 100 mL isopropanol) was added to each well and incubated for 5 min with thorough agitation to solubilize the formazan crystals. An equal volume of the solution was transferred to a 96-well plate and the absorbance was immediately measured using a microplate reader at a wavelength of 570 nm. Results were confirmed by direct measurement of the cells with a standard hemocytometer.

### 2.4. Cell Migration Assay

Cell migration assay was performed using a 48-well Boyden chamber, as described previously [16,34]. Briefly, the upper chamber and lower chamber were separated by an 8-μm pore size polyvinyl pyrrolidone-free polycarbonate membrane (NeuroProbe) coated with collagen. Serum starved HUVECs were added in the upper chamber and M199 with or without VEGF (10 ng/mL) and penfluridol (1 µM) was added to the lower chamber followed by incubation for 6 h at 37 °C. After the incubation, the cells on the upper surface were removed and the ones at the lower surface of the membrane were fixed and stained with hematoxylin. For quantification, the cells were manually counted using a bright-field microscope (Microscoptics, IV-900).

### 2.5. Tube Formation Assay

Tube formation assay was performed with serum-starved HUVECs using growth factor-reduced matrigel, as previously described [16,32,35]. Wells of a 96-well culture plate were carefully coated to avoid bubble formation, with 40 μL/well RGF-Basement membrane extract. After incubation at 37 °C with 5% CO_2_ for 20 min to allow polymerization, 100 µL of starvation medium containing 10^4^ cells was added to each of the respective wells. VEGF (10 ng/mL), penfluridol (1 µM) and SU1498 (5 μΜ) were added in starvation media and incubated for 6 h at 37 °C. At the end of the incubation period, the medium was removed, the cells were fixed and pictures of the wells were captured using an inverted bright-field microscope (Microscoptics, IV-900) connected with a digital camera (AmScope FMA050) at 4X magnification. The pictures were analyzed for the number of nodes, number of junctions and total sprout length and the quantification was performed using the “Angiogenesis analyzer” plug-in [36] in ImageJ 1.6 software (National Institutes of Health).

### 2.6. In Vivo Matrigel Plug and Hemoglobin Assay

In vivo studies were carried out in mice with C57BL/6 background and were maintained according to TTUHSC IACUC-approved protocols in compliance with the Guide for the Care and Use of Laboratory Animals. RGF-Basement membrane extract (BME; 500 μL) combined with or without growth factors (murine bFGF 1.5 ng/μL and murine VEGF 1 ng/μL of BME) were injected subcutaneously into the flank of the mice, as described previously [37]. The next day, the mice were randomly divided into two groups with 6 mice in each group. The test group of mice received 1 mg/kg of penfluridol by oral gavage every day until day 10, and the control mice received vehicle alone, as described previously [27]. On day 11, the mice were euthanized with CO_2_ overdosing and the matrigel plugs were removed aseptically and washed with PBS. Then the plugs were homogenized in PBS using homogenizer and processed for hemoglobin assay with the QuantiChrom^TM^ hemoglobin assay kit, according to the manufacturer’s instructions.

### 2.7. Cancer Cell Paracrine Effect on Angiogenesis

MDA-MB-231 cells were plated on a 6-well culture plate until 80% confluency. Cells were then treated with control (DMSO) and penfluridol (1 μM) for 24 h in starvation medium. After 24 h, the media were removed, washed once with PBS, and starved with M199 starvation medium overnight at 37 °C. The supernatant was then collected, centrifuged at 200× *g* for 5 min, and then used for the cell migration and tube formation experiments described above.

### 2.8. Wound Healing Assay

MDA-MB-231 cells were plated at a density of 0.3 × 10^6^ cells/well and incubated to form a monolayer in 6-well dishes. Once a uniform monolayer was formed, the wound was created by scratching the monolayer with a 1 mL sterile tip. Floating cells were removed by washing the cells with PBS (1X) three times. Further, media was added in all the wells with drug addition, vehicle (DMSO) in the control group, and penfluridol (1 μM) for 24 h in starvation medium. At desired time points, cells were fixed using 10% trichloroacetic acid (TCA) and stained with 0.4% (*w/v*) sulforhodamine B (SRB) dye. The wound was imaged using a bright-field microscope.

### 2.9. Immunoblot Analysis

The cells treated with or without VEGF and penfluridol were lysed on ice in RIPA buffer (10 mmol/L Tris-HCl, 1 mmol/L EDTA, 0.5 mmol/L EGTA, 1% Triton X-100, 0.1% sodium deoxycholate, 0.1% SDS, and 140 mmol/L NaCl), supplemented with protease and phosphatase inhibitors, as previously described [16,31]. Cell lysates were then centrifuged at 13,000 rpm for 10 min at 4 °C and supernatant was mixed with an appropriate volume of SDS loading buffer (5X) and heated to 95–100 °C for 5 min and briefly centrifuged. Equal amounts of protein were subjected to SDS-PAGE, followed by transfer onto a PVDF membrane. After blocking in 5% milk, the membranes were incubated with the appropriate primary antibodies: phospho-VEGF Receptor 2 (Tyr1175) (1:1000), phospho-p38 (Thr180/Tyr182) (1:1000), phospho-ERK (1:2000), phospho-FAK (Tyr397) (1:1000), phospho-Src (Tyr416) (1:1000), phospho-AKT (Ser473) (1:1000), β-actin (1:2000), and β-tubulin (1:2000), followed by incubation with goat anti-rabbit secondary antibody (1:50,000). Immobilon Western Chemiluminescent HRP substrate was used to visualize antigens, according to the manufacturer’s instructions. The protein levels that corresponded to immunoreactive bands were quantified using ImageJ image analysis software (National Institutes of Health).

### 2.10. Statistical Analysis

Each experiment was repeated at least three times, and data analysis was performed using GraphPad Prism version 8.00 for Windows (GraphPad Software, San Diego, CA, USA). Unpaired two-tailed Student’s *t*-tests were performed for statistical significance (NS: not significant, * *p* < 0.05; ** *p* < 0.01; *** *p* < 0.001).

## 3. Results

### 3.1. Identification of Non-Toxic Penfluridol Concentrations

**Figure 1:**
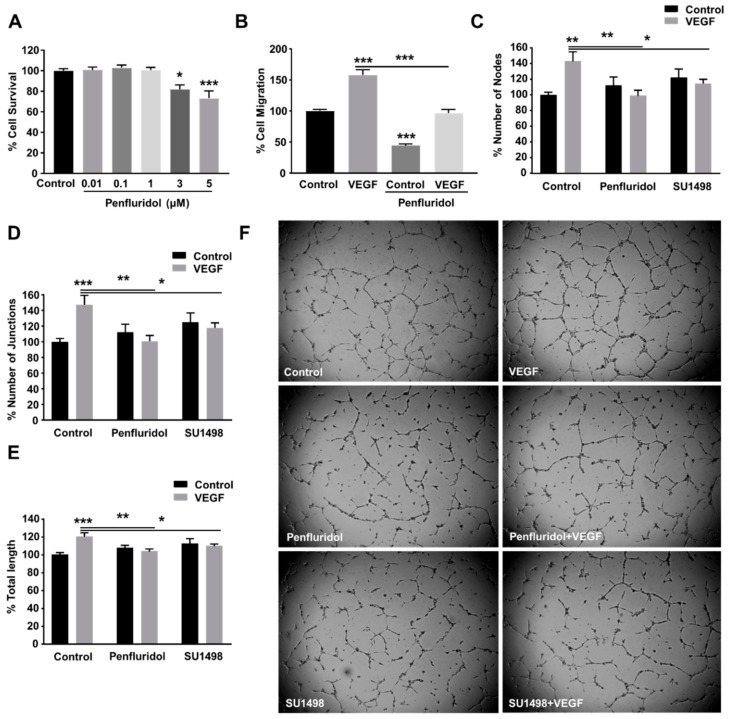
Effect of low concentration of penfluridol on endothelial cell functions. (**A**) Quantification of endothelial cell survival after dose response of penfluridol treatment (*n* = 4). (**B**–**C**) Quantification of VEGF-induced cell migration (*n* = 3) (**B**) and tube formation (*n* = 4), assessed by number of nodes (**C**), number of junctions (**D**) and total length (**E**), in the presence or absence of 1 μΜ penfluridol or 5 μΜ SU1498. (**F**) Representative images of endothelial cell sprouts in the presence of VEGF, penfluridol, or combination thereof. * *p* < 0.05; ** *p* < 0.01; *** *p* < 0.001.

Previous studies have shown that penfluridol suppresses the growth of breast cancer, pancreatic cancer, and glioblastoma cells in vitro by various mechanisms [27,28,29]. In our study, we wanted to evaluate whether a low concentration of penfluridol affects the angiogenic potential of endothelial cells. To perform angiogenesis experiments, we first aimed to identify the maximum concentration at which penfluridol does not exert any cytotoxicity on endothelial cells. For this purpose, we performed an MTT cytotoxicity assay using different concentrations of penfluridol (Figure 1A) for 48 h in human umbilical vein endothelial cells (HUVECs). We identified that penfluridol does not affect endothelial cell viability in concentrations up to 1 µM, while 20% and 40% of cell death occurred after 48 h treatment with 3 and 5 µM of penfluridol, respectively. Therefore, the penfluridol dose of 1 µM was considered safe for HUVECs and was chosen to be used for further angiogenesis experiments.

### 3.2. Low Concentration of Penfluridol Inhibits Endothelial Cell Migration and Tube Formation In Vitro

Vascular endothelial growth factor (VEGF) is one of the most upregulated pro-angiogenic growth factors in pathological angiogenesis and is a well-described key regulator of tumor angiogenesis. Therefore, the most successful anti-angiogenic therapies to date target VEGF or the downstream signaling pathway [11,38]. VEGF was also selected in our study to induce angiogenesis in vitro and evaluate the effect of penfluridol on VEGF-induced endothelial cell migration and tube formation. We identified 10 ng/mL as the optimal VEGF concentration for the induction of HUVEC migration and tube formation (not shown) and selected that dose for future experiments. Penfluridol treatment (1 μM) for 24 h inhibited the basal migratory activity of HUVECs by ~50% and completely abrogated VEGF-induced endothelial cell migration (Figure 1B). The capillary-like tube formation on matrigel is considered a reliable quantifiable parameter of in vitro angiogenesis [16,35]. We assessed the effect of penfluridol on VEGF-induced tube formation (Figure 1C–F) and compared it with working concentration (5 μΜ) of SU1498 [39], a selective inhibitor of VEGFR2 tyrosine kinase [40]. Similarly to SU1498, penfluridol significantly abrogated VEGF-induced tube formation in vitro, assessed by the number of nodes (Figure 1C,F), number of junctions (Figure 1D,F), and total tube length (Figure 1E,F), confirming its anti-angiogenic in vitro potential.

### 3.3. Low Dose of Penfluridol Blocks In Vivo Angiogenesis

**Figure 2:**
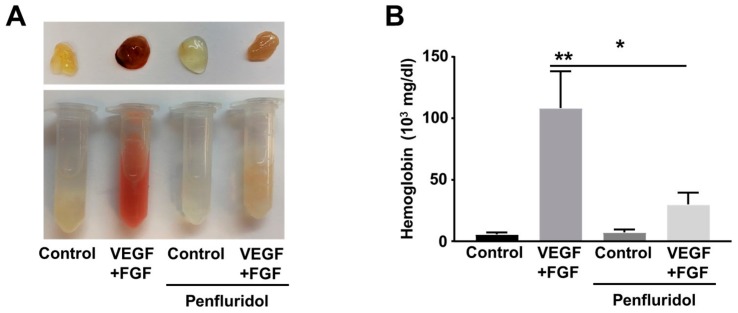
Effect of low dose of penfluridol treatment on VEGF- and FGF-induced angiogenesis in vivo (*n* = 3). Representative images (**A**) and quantification of hemoglobin levels (**B**) of matrigel plugs treated with VEGF + bFGF, penfluridol or combinations thereof. * *p* < 0.05; ** *p* < 0.01.

To identify the effect of penfluridol on in vivo angiogenesis, we performed the matrigel plug angiogenesis assay in immune-competent mice. Blockade of the VEGF signaling pathway by anti-angiogenic treatment often triggers upregulation and compensatory activity of other growth factors, from which bFGF is the best known [41,42,43]. In our hands, VEGF and bFGF combination leads to higher angiogenic effects than each factor individually (not shown). To validate our in vitro findings and identify potential effect of penfluridol on combined VEGF- and bFGF-induced angiogenesis in vivo, we implanted matrigel plugs containing both VEGF and bFGF in flanks of C57B/L6 mice. The day after matrigel implantation, the mice were randomly separated in two groups and treated orally with low dose penfluridol (1 mg/kg) or vehicle daily. Eleven days after implantation, the plugs were isolated, and vascularization was qualitatively evaluated by the red color of the plugs, demonstrating blood circulation (Figure 2A), and quantitatively by estimation of hemoglobin concentration (Figure 2B). Presence of growth factors induced more than 10-fold increase in hemoglobin levels in vehicle-treated mice. However, in penfluridol-treated mice, matrigel plugs containing growth factors were paler and presented significantly lower (~75%) hemoglobin levels compared to the growth factor-containing plugs of the vehicle-treated mice. Therefore, from both parameters, it was obvious that penfluridol potently inhibited growth factor-induced angiogenesis in vivo (Figure 2).

### 3.4. Low Concentration of Penfluridol Affects the Downstream VEGF-Signaling Pathway in Endothelial Cells

**Figure 3:**
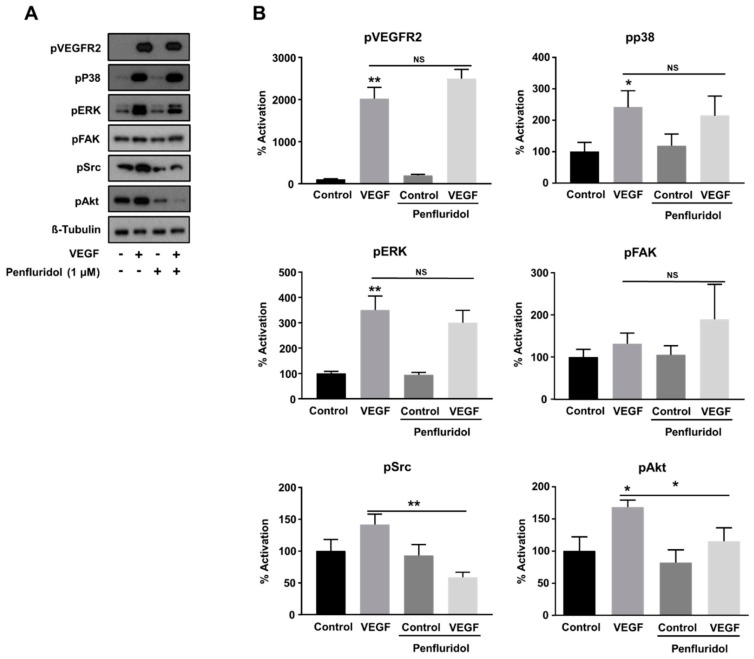
Effect of low concentration of penfluridol (1 μΜ) on VEGF-induced signaling pathways in endothelial cells. Representative Western blot images (**A**) and quantification (**B**) of the phosphorylation levels of VEGFR2, p38, ERK, FAK, Src and AKT, normalized with tubulin levels (*n* = 5). * *p* < 0.05; ** *p* < 0.01; NS = not significant.

VEGF regulates endothelial cell functions through activation of its cognitive receptor, VEGFR2, and the activation of the following downstream effectors in the endothelial cells: (i) ERK, (ii) p38, (iii) focal adhesion kinase (FAK), and (iv) Akt, through distinct signaling pathways [44,45]. Since penfluridol inhibited VEGF-induced angiogenesis in vitro and in vivo, we wanted to explore the key signaling molecules potentially responsible for this inhibition. Penfluridol did not affect VEGF-induced VEGFR2 phosphorylation (Figure 3A,B) and similarly, VEGF-induced p38 and ERK activation were not abrogated by penfluridol treatment (Figure 3A,B), nor a striking effect was observed on FAK activity either. However, Akt activation was inhibited (~60% from VEGF induction) upon penfluridol treatment, as well as VEGF-induced Src activation (below the control levels) (Figure 3A,B), demonstrating that the inhibition of the Src and Akt signaling pathways is sufficient to block VEGF activity.

### 3.5. Effect of Low Concentration of Penfluridol on Breast Cancer Cell Functions and Basal Src Activation

**Figure 4:**
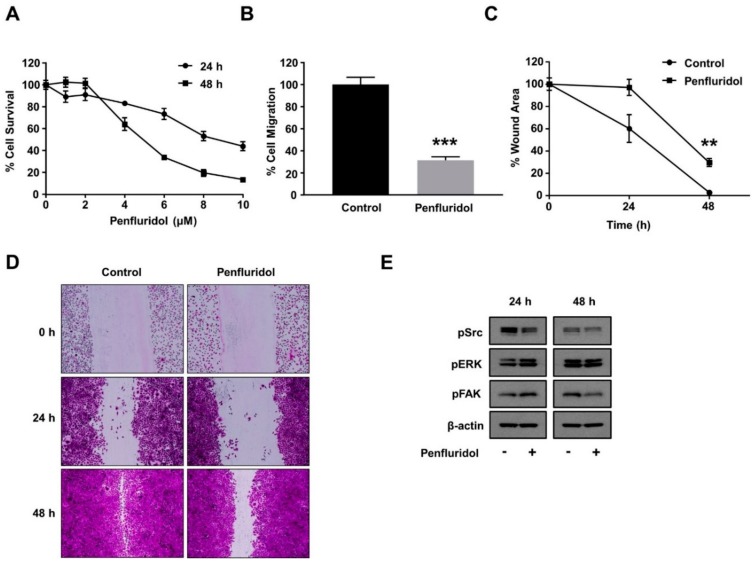
Effect of low concentration of penfluridol on breast cancer cell functions. (**A**) Quantification of breast cancer cell survival after dose response of penfluridol treatment (*n* = 3). (**B**) Quantification of spontaneous cancer cell migration in the presence of vehicle or penfluridol treatment (*n* = 3). (C,D) Quantification (**C**) and representative images (**D**) of cancer cell in vitro wound healing assay in the presence of vehicle or penfluridol treatment (*n* = 3). (**E**) Representative images demonstrating phosphorylation levels of Src, ERK and FAK, and actin levels (*n* = 3). ** *p* < 0.01; *** *p* < 0.001.

Tumor angiogenesis goes hand-in-hand with tumor progression, and both cancer and endothelial cells are exposed to similar penfluridol levels during clinical use. Therefore, it is important to evaluate the effect of the same low dose of penfluridol dose on cancer cell viability, basic cancer cell functions, and important signaling mediators. A low dose of penfluridol did not affect the survival of the triple-negative breast cancer cell line MDA-MB-231 at 24 and 48 h (Figure 4A); however, it significantly inhibited (>60%) breast cancer cell migration (Figure 4B). In a similar manner, penfluridol treatment abrogated the wound closure efficiency of the breast cancer cells in a wound healing assay (Figure 4C,D). The wound healing efficiency is highly dependent on the combined effect of the migration and proliferation efficiency of the tested cells, and since the proliferation efficiency was not significantly affected (Figure 4A), this further demonstrates the significant inhibition low dose of penfluridol causes on breast cancer cell migration. To investigate the molecular basis of this inhibition, the basal activation levels of important downstream signaling pathways were evaluated. Contrary to the endothelial cells, the basal activation levels of FAK and ERK were not inhibited, but Src basal activation was inhibited in both 24 and 48 h incubation periods (Figure 4E).

### 3.6. The Effect of Low Concentration of Penfluridol on Endothelial and Breast Cancer Cells Are Not Dependent on Cancer Cell-Derived Paracrine Effect on Endothelial Cells

**Figure 5:**
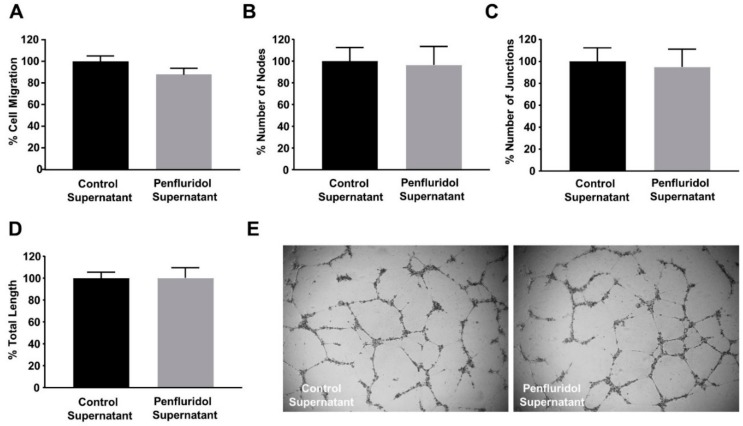
Treatment of breast cancer cells with low concentration of penfluridol does not alter their paracrine communication with endothelial cells. (**A**) Quantification of spontaneous endothelial cell migration in the presence of starvation supernatant from cancer cells previously treated with vehicle or penfluridol (*n* = 3). (**B**–**E**) Quantification (**B**–**D**) and representative images (**E**) of spontaneous tube formation of endothelial cells in the presence of starvation supernatant from cancer cells previously treated with vehicle or penfluridol (*n* = 3), assessed by number of nodes (**B**), number of sprouts (**C**) and total length (**D**).

The co-existence of tumor and stroma cells in the tumor and peritumoral area is known to induce the development of paracrine communication mechanisms. We wanted to identify whether a low dose of penfluridol treatment on breast cancer cells affects their secretome and, consequently, their paracrine communication with endothelial cells. For this, cancer cells were treated with penfluridol or vehicle in starvation media for 24 h, the medium was replaced with endothelial-specific starvation medium overnight and the angiogenic effect of the cancer cell-derived supernatant was assessed on endothelial cell functions. As shown in Figure 5, penfluridol treatment did not affect the migratory (Figure 5A) nor the angiogenic potential (Figure 5B–E) of endothelial cells, demonstrating that the effect of a low dose of penfluridol on each cell type is due to the direct effect of the compound and is not affected by paracrine signaling of diverse adjacent cell types.

## 4. Discussion

Penfluridol is a long acting oral antipsychotic agent for schizophrenia treatment, widely used in the clinic since 1970 [46]. It has been recently demonstrated to have potent anti-cancer activity for breast cancer [27,30,47], pancreatic cancer [28,48] and glioblastoma [29,49]. A series of characteristics of this drug, such as its in vivo stability, allowing weekly administration to schizophrenia patients, the high potency to cross the blood–brain-barrier, and the anticancer activity, highlight the potential for repurposing this drug for cancer treatment.

The higher genetic stability of the tumor microenvironment, contrary to the adjacent genetically unstable tumor cells, provides great potential for anticancer treatments. Penfluridol has been shown to affect the tumor microenvironment by suppressing the myeloid-derived suppressor cells (MDSCs) and thus elevating the M1 macrophage levels in glioblastoma [49]. Here, we show that a low dose of penfluridol blocks another aspect of tumor microenvironment, angiogenesis. A low dose of penfluridol blocked VEGF-induced endothelial cell migration and tube formation in vitro, in a similar extent to VEGFR2 inhibitors, and angiogenesis in vivo. At the molecular level, penfluridol inhibits VEGF-induced Src and Akt phosphorylation, while VEGFR2, p38 and ERK activation levels are not affected. VEGFR2 phosphorylation activates several signaling pathways that regulate angiogenesis. Focal adhesion kinase (FAK) is a cytoplasmic tyrosine kinase which regulates the cell functions of both tumor cells, as well as other cell types in the tumor microenvironment, including the endothelial ones [44,50]. Src activation is required for VEGF-induced angiogenesis and permeability [51,52]. Src activation initiates multiple downstream signaling pathways via phosphorylation of different proteins that eventually lead to important cellular responses such as cell survival, proliferation, migration and invasion, and in the endothelial context, the central role of Src complex in angiogenesis is well established [53,54,55,56]. Therefore, novel anticancer agents that can target Src activity could provide promising treatment options to inhibit angiogenesis and thus cancer progression [57]. The fact that the VEGFR2 phosphorylation levels are not affected denotes that penfluridol exerts its anti-angiogenic activity not by blocking VEGF interaction to VEGFR2, but instead by a yet unidentified mechanism, affecting Src and Akt phosphorylation. It would be interesting to test in future studies whether penfluridol blocks Src and Akt activation driven by other growth factors as well, as that would signify a universal anti-angiogenic mechanism.

Low doses of penfluridol also inhibited breast cancer cell migration, but through a distinct mechanism than the ones reported with the higher, cancer cytotoxic doses [27]. This leads to one of the important elements of this study, which was the low dose of penfluridol in the in vitro, and also in the in vivo experiments. The penfluridol dose prescribed for chronic schizophrenia and similar psychotic disorders ranges from 60 to 140 mg weekly, with most studies having an average dose of 80 mg [23,24,58]. The oral administration of 1 mg/kg dose in mice corresponds to only 39 mg/week of human dose, which, according to allometric dose scaling based on body surface area [59], is half of the average weekly dose prescribed to schizophrenia patients. This highlights the potential effects clinically administered penfluridol could have in angiogenic mechanisms of the human body. It has been reported that the overall cancer incidence rate among schizophrenia patients is significantly lower than that of the general population [60,61,62], and the influence antipsychotic drugs may have on cancer incidence has also been reported, although penfluridol was not included in that systematic review [63]. Similarly, angiogenesis has been reported to be the missing link in this reverse correlation; however, no supporting data have been presented to date [64]. Given the present findings, it would be interesting to correlate penfluridol use with cancer incidence and outcome of anti-cancer treatment of the prescribed patients.

Overall, this study demonstrates that low, clinically-relevant doses of penfluridol administration affect the cellular functions of both cancer and endothelial cells through direct but independent mechanisms; thus also affecting the tumor microenvironment and blocking growth factor-induced angiogenesis.
